# Algal Bio-Stimulants Enhance Salt Tolerance in Common Bean: Dissecting Morphological, Physiological, and Genetic Mechanisms for Stress Adaptation

**DOI:** 10.3390/plants12213714

**Published:** 2023-10-29

**Authors:** Hoda H. Senousy, Yousef Alhaj Hamoud, Abdelghafar M. Abu-Elsaoud, Omar Mahmoud Al zoubi, Nessreen F. Abdelbaky, Muhammad Zia-ur-Rehman, Muhammad Usman, Mona H. Soliman

**Affiliations:** 1 Botany and Microbiology Department, Faculty of Science, Cairo University, Giza 12613, Egypt; hsenousy@sci.cu.edu.eg (H.H.S.);; 2 College of Hydrology and Water Recourses, Hohai University, Nanjing 210098, China; 3 Department of Biology, College of Science, Imam Muhammad Ibn Saud Islamic University (IMSIU), Riyadh 11623, Saudi Arabia; 4 Department of Botany and Microbiology, Faculty of Science, Suez Canal University, Ismailia 41522, Egypt; 5 Biology Department, Faculty of Science Yanbu, Taibah University, Yanbu El-Bahr 46423, Saudi Arabia; 6 Biology Department, Faculty of Science, Taibah University, Al-Sharm, Yanbu El-Bahr, Yanbu 46429, Saudi Arabia; 7 Institute of Soil and Environmental Sciences, University of Agriculture, Faisalabad 38000, Pakistan

**Keywords:** antioxidants, *Chlorella vulgaris*, *Dunaliella salina*, osmolytes, *Phaseolus vulgaris*, photosynthesis, salinity

## Abstract

Salinity adversely affects the plant’s morphological characteristics, but the utilization of aqueous algal extracts (AE) ameliorates this negative impact. In this study, the application of AE derived from *Chlorella vulgaris* and *Dunaliella salina* strains effectively reversed the decline in biomass allocation and water relations, both in normal and salt-stressed conditions. The simultaneous application of both extracts in salt-affected soil notably enhanced key parameters, such as chlorophyll content (15%), carotene content (1%), photosynthesis (25%), stomatal conductance (7%), and transpiration rate (23%), surpassing those observed in the application of both AE in salt-affected as compared to salinity stress control. Moreover, the AE treatments effectively mitigated lipid peroxidation and electrolyte leakage induced by salinity stress. The application of AE led to an increase in GB (6%) and the total concentration of free amino acids (47%) by comparing with salt-affected control. Additionally, salinity stress resulted in an elevation of antioxidant enzyme activities, including superoxide dismutase, ascorbate peroxidase, catalase, and glutathione reductase. Notably, the AE treatments significantly boosted the activity of these antioxidant enzymes under salinity conditions. Furthermore, salinity reduced mineral contents, but the application of AE effectively counteracted this decline, leading to increased mineral levels. In conclusion, the application of aqueous algal extracts, specifically those obtained from *Chlorella vulgaris* and *Dunaliella salina* strains, demonstrated significant efficacy in alleviating salinity-induced stress in *Phaseolus vulgaris* plants.

## 1. Introduction

Population growth poses a challenge to natural resources and agricultural productivity, exacerbating global food scarcity [1,2]. The scarcity of resources is compounded by global environmental stresses, such as heat, cold, drought, and salinity, affecting plant growth, production, yield, and food quality and impeding efforts to alleviate hunger [3,4,5,6]. Soil salinization presents a pressing concern, particularly for cultivating economically significant crops [7,8,9,10,11,12]. Investments in adaptation strategies for climate-resilient agriculture aim to mitigate climate-induced threats to food security [13,14,15]. Ionic, osmotic, and oxidative stresses collectively contribute to the harmful effects of salinity on plant growth [16,17]. Plants respond to salinity stress by preserving their cellular osmotic potential, underscoring the connection between environmental change and survival [18]. Salinity triggers the production of reactive oxygen species (ROS), such as hydrogen peroxide (H_2_O_2_) and superoxide radicals in plants, disrupting ion balance. Activation of stress signaling networks prompts subsequent physiological and biochemical responses to salinity stress [19]. Elevated oxidative stress leads to reduced transpiration rates [20,21], impaired water absorption, altered nutrient uptake dynamics, diminished chlorophyll production [22,23,24], and compromised growth and yield [25,26,27]. To thrive under salinity stress, plants have developed morphological, physiological, and metabolic adaptations [28]. Effective management of antioxidant defense, whether enzymatic or non-enzymatic [27,29,30,31], is crucial for mitigating salt-induced oxidative stress. Additionally, the accumulation of osmoprotectants plays a pivotal supportive role in counteracting salt-induced changes [32,33]. Addressing salinity-related challenges involves diverse approaches, including natural extracts and fertilizers, necessitating informed decision-making for effective solutions.

Enhancing agricultural crop productivity in challenging conditions is effectively achieved through the application of beneficial bio-stimulants, such as biofertilizers, mycorrhizas, algal extracts (AE), and organic matter [34,35]. Bio-stimulants consist of sustainable and eco-friendly bioactive compounds promoting plant growth and development [10,36]. For fortification and recovery, substances, such as proline, amides, ᵞ-aminobutyric acid (GABA), and glycine betaine (GB), can be integrated into bio-stimulants [37]. These bio-stimulants regulate physiological processes to optimize plant productivity under normal and stressful conditions, enhancing soil nutrient uptake and nutrient quality [38]. Algae and arbuscular mycorrhizal fungus extracts have demonstrated increased productivity, yield, and root and shoot biomass [39,40]. Bioactive metabolites, such as osmolytes and mineral nutrients, target critical pathways, reducing salinity stress impact and safeguarding tissue turgor and water potential [41,42]. Utilizing bio-stimulants derived from algae has emerged as an innovative approach to enhance plant responsiveness to environmental challenges, including salinity [37,43]. Notably, three Egyptian seaweeds (*Ulva fasciata*, *Cystoseira compressa*, and *Laurencia obtusa*) offer liquid extracts that serve as effective bio-stimulants, elevating salt tolerance in various plants, such as *Zea mays* and *Vigna sinensis* [44]. Previous experiments have demonstrated a practical strategy to enhance nutrient uptake, growth, and salt tolerance in tomato plants. This approach involves using formulations of microalgae-cyanobacteria extracts, including *Arthrospira maximum*, *Chlorella ellipsoidea*, *Aphanothece* sp., and *Dunaliella salina* [45]. Researchers have investigated the impact of *Dunaliella salina* under salt stress on *Cucurbita pepo* L. cv. Mabrouka [46]. The findings indicate that the application of *D*. *salina* enhances the plants’ ability to withstand salt stress, presenting a promising avenue for further research.

*Phaseolus vulgaris* L., commonly known as the common bean, is a vital legume cultivated worldwide for its edible seeds and pods. These beans serve as a fundamental source of proteins, essential minerals (Fe and Zn), and vitamins in many human diets [47]. Notably, bean grains are rich in vitamins, minerals, and proteins [48]. This warm-season, annual herbaceous crop is susceptible to environmental fluctuations [49]. To ensure a steady supply of bean grains, enhancing their performance in saline conditions is crucial. This requires innovative and eco-friendly methods to bolster their resilience to salinity stress. As such, we aimed to investigate the effects of microalgae extracts on bean plant responses to salt stress, nutrient uptake, and vegetative growth in saline environments. Our study seeks to reveal: (a) the advantageous impact of algal extracts on growth and biomass production in common bean, (b) the enduring effects of salinity on pathways influenced by salinity stress, (c) the intricate interactions of biochemical and genetic factors underlying salinity stress tolerance in common bean, and (d) the favorable influence of algal extracts on plant exposure and performance.

## 2. Results

### 2.1. Biochemical Analyses of Algal Species

During the present study, two types of algae were utilized. The research involved the examination of extracts obtained from *C. vulgaris* (referred to as EXc) and *D. salina* (referred to as EXd) in terms of their biochemical properties. The outcomes of this analysis are presented in Table 1. In terms of the total protein content, the average measurement for *C. vulgaris* was 0.32 ± 0.01, whereas for *D. salina*, it was 0.16 ± 0.01. A notable distinction between these two species was observed. The research delved further into detailed biochemical assessments, encompassing various factors such as total protein, total sugar content, proline, GB, total N, total P, total K, Mg, and Na. These results are comprehensively documented in Table 1.

### 2.2. Plant Growth Response under Salts Stress and Algal Extracts Application

**Figure 1:**
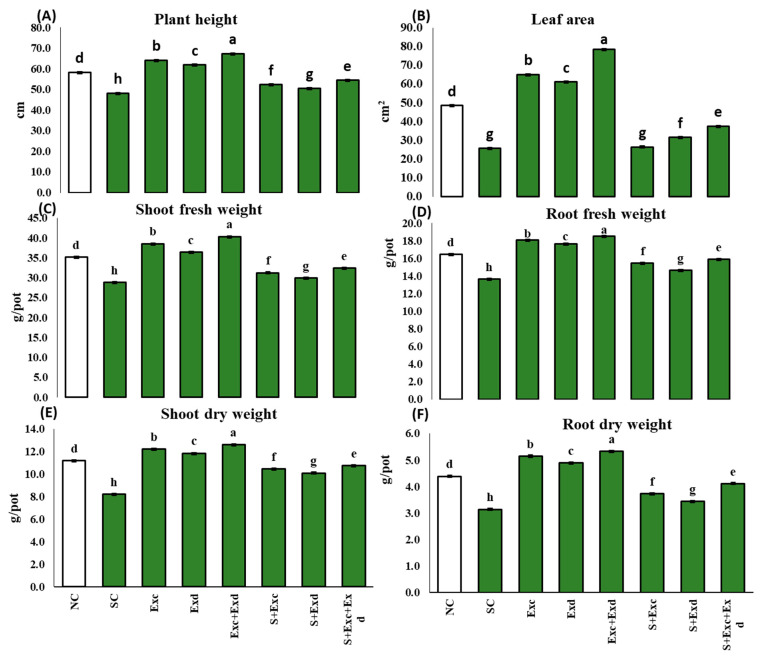
Effect of *Chlorella vulgaris* and *Dunaliella salina* extracts on (**A**) plant height, (**B**) leaf area, (**C**) shoot fresh weight, (**D**) root fresh weight, (**E**) shoot dry weight, (**F**) root dry weight in common bean plants under normal and saline condition soils. On *x*-axis NC; normal soil control, SC; salt-affected soil control, Exc; extract of *Chlorella vulgaris*, Exd; extract of *Dunaliella salina*, Exc + Exd; combined application of *Chlorella vulgaris* and *Dunaliella salina* in normal soil, S + Exc; application of *Chlorella vulgaris* in salt-affected soil, S + Exd; application of *Dunaliella salina* in salt-affected soil, S + Exc + Exd; combined application of *Chlorella vulgaris* and *Dunaliella salina* in salt-affected soil. Bar chart presenting mean values of different treatments, error bars indicating standard deviation, and different lettering on the bars highlighting the significance difference among the applied treatments at a 5% level of significance by applying Duncan’s Multiple Range Test (DMRT).

**Figure 2:**
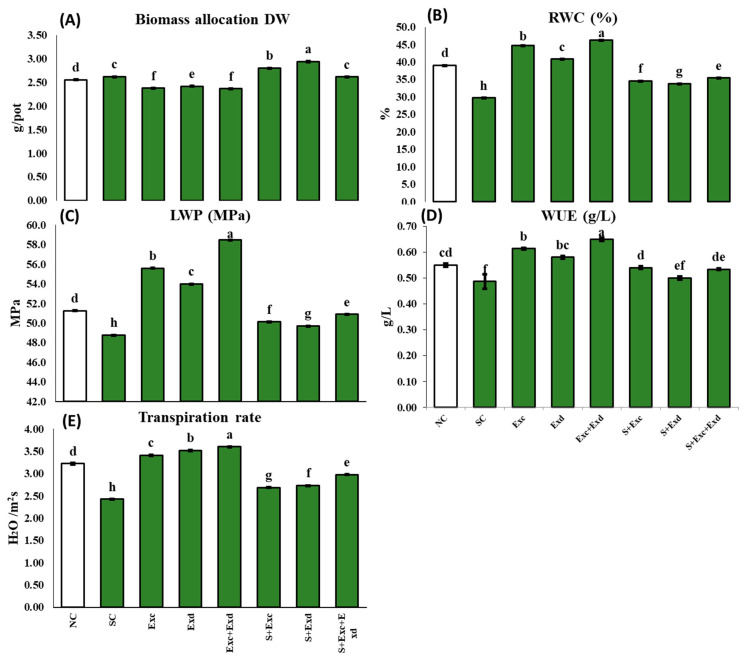
Effect of *Chlorella vulgaris* and *Dunaliella salina* extracts on (**A**) biomass allocation (Shoot: Root ratio), (**B**) relative water content, (**C**) Leaf water potential (LWP; MPa), (**D**) WUE, and (**E**) The transpiration rate (Tr, mmol m^−2^ s^−1^) in common bean plants under normal and saline condition soils. On *x*-axis NC; normal soil control, SC; salt-affected soil control, Exc; extract of *Chlorella vulgaris*, Exd; extract of *Dunaliella salina*, Exc + Exd; combined application of *Chlorella vulgaris* and *Dunaliella salina* in normal soil, S + Exc; application of *Chlorella vulgaris* in salt-affected soil, S + Exd; application of *Dunaliella salina* in salt-affected soil, S + Exc + Exd; combined application of *Chlorella vulgaris* and *Dunaliella salina* in salt-affected soil. Bar chart presenting mean values of different treatments, error bars indicating standard deviation, and different lettering on the bars highlighting the significance difference among the applied treatments at a 5% level of significance by applying Duncan’s Multiple Range Test (DMRT).

The normal control treatment exhibited the maximum plant height (cm) of 58.3 ± 0.01. This height notably diminished the salinity stress control at 48.2 ± 0.01 cm. Upon exogenous application of EXc and EXd, the plant height was significantly incremented, reaching 64.2 and 61.9 cm, respectively, in comparison to the salt-affected control plants (Figure 1). Under salinity stress, the leaf area decreased by 25.99 ± 0.02 mm^2^. Conversely, the application of EXc and EXd led to a substantial increase in leaf area, reaching averages of 65.2 and 61.2 mm^2^, respectively. The fresh weight of roots and shoots in salinity-exposed plants measured 28.8 ± 0.01 and 13.6 ± 0.012 g/plant, respectively. The application of EXc and EXd led to significant enhancements in shoot fresh weight, reaching averages of 38.6 ± 0.01 and 36.4 ± 0.01 g/plant, respectively, while plants treated with both extracts (EXc + ECd) recorded a weight of shoots and roots as 40.3 and 18.5 g/plant. Examining shoot dry weight, the highest dry weight was recorded in plants treated with a combination of EXc and EXd (12.6 ± 0.015), followed by EXc (12.2 ± 0.010) and EXd (11.9 ± 0.010). Salinity-stressed plants exhibited the lowest shoot dry weight (8.2 ± 0.01). Remarkably, treatment with EXc, EXd, or combined application significantly enhanced shoot dry weight under salinity stress conditions. Similar trends were observed for shoot and root dry weight, with the lowest values in salinity-stressed plants and the highest in plants treated with EXc and EXd. The application of the two extracts notably improved shoot and root dry weight under salinity stress (Figure 1). Biomass allocation, represented by the ratio of shoot to root biomass, did not exhibit substantial variations across treatments. Relative water content (RWC) reached its peak in plants treated with both extractants. In contrast, the lowest RWC was recorded in salinity-stressed plants (29.7 ± 0.010). The application of EXc, EXd, or in combined form led to an improvement in RWC for stressed plants (Figure 2).

### 2.3. Water Relation and Photosynthetic Response under Salinity Stress and Algal Extracts Application

The salinity-exposed plants exhibited a significantly lower leaf water potential of 48.8 ± 0.021 MPa, which increased to 50.1 ± 0.010, 49.7 ± 0.015, and 50.9 ± 0.010 MPa in response to EXc, EXd, and EXc + EXd treatments, respectively. The application of both extracts (EXc and EXd) led to the highest leaf water potential of 58.5 ± 0.021 MPa. Similarly, plant water use efficiency (WUE) reached its peak at 0.65 ± 0.010 g/L in plants treated with both extracts. However, there were negligible changes in WUE among the other treatments (Figure 2).

**Figure 3:**
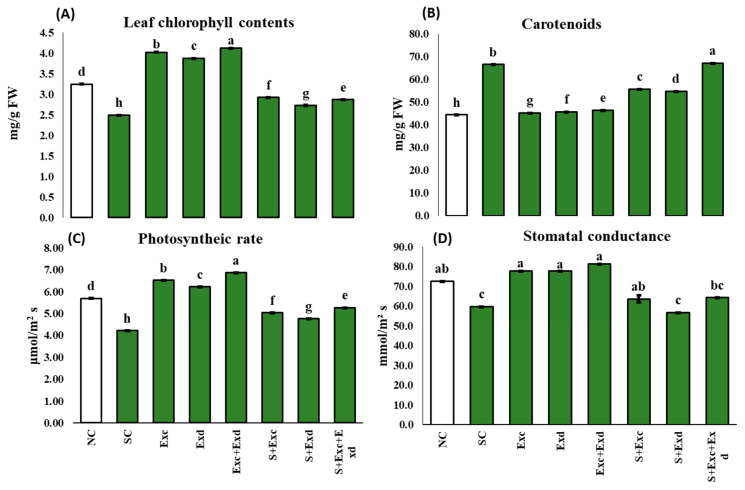
Effect of *Chlorella vulgaris* and *Dunaliella salina* extracts on (**A**) Leaf chlorophyll contents, (**B**) Carotenoids, (**C**) Photosynthetic rate (Pn), (**D**) Leaf stomatal conductance (Gs) in common bean plants under normal and saline condition soils. On *x*-axis NC; normal soil control, SC; salt-affected soil control, Exc; extract of *Chlorella vulgaris*, Exd; extract of *Dunaliella salina*, Exc + Exd; combined application of *Chlorella vulgaris* and *Dunaliella salina* in normal soil, S + Exc; application of *Chlorella vulgaris* in salt-affected soil, S + Exd; application of *Dunaliella salina* in salt-affected soil, S + Exc + Exd; combined application of *Chlorella vulgaris* and *Dunaliella salina* in salt-affected soil. Bar chart presenting mean values of different treatments, error bars indicating standard deviation, and different lettering on the bars highlighting the significance difference among the applied treatments at a 5% level of significance by applying Duncan’s Multiple Range Test (DMRT).

Regarding stomatal conductance (μmol/m^2^s), the highest levels were observed with the application of both extracts (81.4 ± 0.02), followed by EXc (77.6 ± 0.055) and EXd (77.6 ± 0.055). Salinity-stressed plants displayed an average of 59.8 ± 0.01 μmol/m^2^s, which notably improved upon the application of EXc (63.6 ± 2.9 μmol/m^2^s), EXd (56.7 ± 0.015 μmol/m^2^s), and both extracts in combination (64.2 ± 0.01 μmol/m^2^s) (Figure 3). The photosynthetic rate (Pn) witnessed a significant decrease under salinity stress, reaching a level of 4.22 ± 0.010 μmol H₂O/m^2^s. The highest Pn (25%) was recorded following treatment with a combination of both extracts under salinity stress, while EXd slightly enhanced Pn (13%) under salinity stress, EXc led to a notable increment in Pn (19%) as compared to control (Figure 3). Leaf chlorophyll content (mg/g FW) was maximum in both extracts, which was 4.1 ± 0.01. Conversely, the lowest significant leaf chlorophyll content was observed in plants subjected to salinity stress, measuring 2.5 ± 0.010 mg/g FW. The application of AE treatments significantly elevated the total chlorophyll content in plants under stressed conditions. A two-way ANOVA indicated highly significant effects of salinity stress (*p* < 0.001), EXc (*p* < 0.001), EXd (*p* < 0.001), and their interactions (*p* < 0.001) (Figure 3). Similarly, a two-way ANOVA revealed significant variations in the amount of leaf carotenoids (mg g^−^¹ FW) due to salinity (*p* < 0.001), EXc (*p* < 0.001), EXd (*p* < 0.001), the interaction between salinity and EXc (*p* < 0.001), and the interactions of salinity with both extracts 1 and 2 (*p* < 0.001) (Figure 3).

### 2.4. Oxidative Stress Indicators under Salinity Stress and Algal Extracts Application

**Figure 4:**
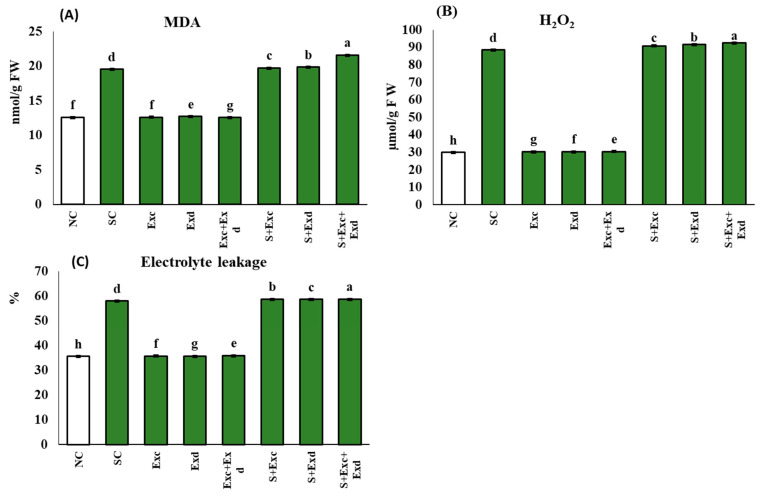
Effect of *Chlorella vulgaris* and *Dunaliella salina* extracts on (**A**) Cellular lipid peroxidation (MDA; nmol g^−1^ FW), (**B**) Hydrogen peroxide (H_2_O_2_; µmol g^−1^ FW), (**C**) Electrolyte leakage (EL; %) in common bean plants under normal and saline condition soils. On *x*-axis NC; normal soil control, SC; salt-affected soil control, Exc; extract of *Chlorella vulgaris*, Exd; extract of *Dunaliella salina*, Exc + Exd; combined application of *Chlorella vulgaris* and *Dunaliella salina* in normal soil, S + Exc; application of *Chlorella vulgaris* in salt-affected soil, S + Exd; application of *Dunaliella salina* in salt-affected soil, S + Exc + Exd; combined application of *Chlorella vulgaris* and *Dunaliella salina* in salt-affected soil. Bar chart presenting mean values of different treatments, error bars indicating standard deviation, and different lettering on the bars highlighting the significance difference among the applied treatments at a 5% level of significance by applying Duncan’s Multiple Range Test (DMRT).

The oxidative stress was assessed in terms of cellular accumulation of MDA, H_2_O_2_, and Electrolyte leakage (%). Oxidative damage was presented in Figure 4A–C. The MDA (nmol/g FW) recorded an average of 21.58 nmol/g FW in EXc + EXd was applied in salt-affected soil. The trend of applied treatments remained as EXc + EXd (10%) > EXd (2%) > EXc (1%) in salinity-stressed soil while as EXc + EXd (−36%) < EXd (−35%) = EXc (−35%) in normal soil as compared to salt-affected control treatment. The same was noticed in the hydrogen peroxide accumulation level, where the highest values were recorded in the combined application of EXc and EXd under salinity stress which was 92.6 µmol/g FW, while the trend of applied treatments remained as EXc + EXd (5%) > EXd (4%) > EXc (3%) in salt-affected soil. Under normal soil conditions, the applied treatments remained as EXc + EXd (−66%) = EXc (−66%) = EXd (−66%) as compared to salinity stressed control. The electrolyte leakage was significantly affected by the application of EXc and EXd alone and combined application in normal and salt-affected soil. The treatments remained as EXc + EXd (58.7%) = EXc (58.7%) > EXd (58.5%) > in salinity-stressed soil while under normal soil conditions EXc + Exd (35.8%) > Exc (35.7%) = Exd (35.7%).

### 2.5. Osmolytes Response under Salinity Stress and Algal Extracts Application

**Figure 5:**
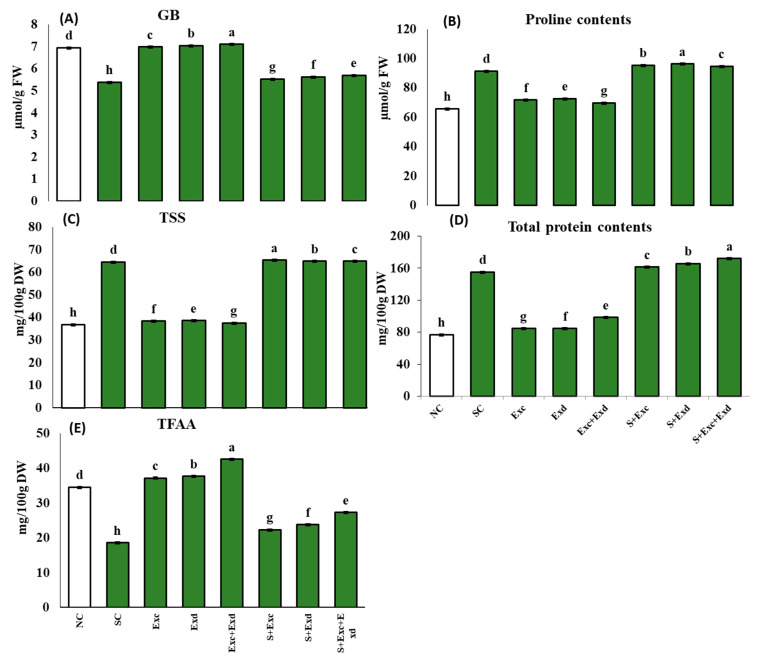
Effect of *Chlorella vulgaris* and *Dunaliella salina* extracts on (**A**) Glycine betaine (GB; µmol/g FW), (**B**) Proline content (µmol/g FW), (**C**) total soluble sugars (mg/100 g DW), (**D**) Total protein content (mg/100 g DW), (**E**) total free amino acids (TFAA, mg/100 g DW) in common bean plants under normal and saline condition soils. On *x*-axis NC; normal soil control, SC; salt-affected soil control, Exc; extract of *Chlorella vulgaris*, Exd; extract of *Dunaliella salina*, Exc + Exd; combined application of *Chlorella vulgaris* and *Dunaliella salina* in normal soil, S + Exc; application of *Chlorella vulgaris* in salt-affected soil, S + Exd; application of *Dunaliella salina* in salt-affected soil, S + Exc + Exd; combined application of *Chlorella vulgaris* and *Dunaliella salina* in salt-affected soil. Bar chart presenting mean values of different treatments, error bars indicating standard deviation, and different lettering on the bars highlighting the significance difference among the applied treatments at a 5% level of significance by applying Duncan’s Multiple Range Test (DMRT).

Various important cellular molecules, including glycine betaine, proline content, total soluble sugars, total protein content, and total free amino acids, were significantly affected by salinity stress and treated with EXc and EXd. Salinity stress reduced the glycine betaine (5.38 ± 0.015 µmol/g FW), increased after the application of EXc and EXd, and combined applicaion to record a level of 6.99 ± 0.015, 7.05 ± 0.010, and 7.11 ± 0.010. The trend of applied treatments for proline contents remained as EXd (5%) > EXc + EXd (4%) = EXd (4%) in salt-affected soil, while, in normal soil conditions, the trend was EXc + EXd (−24%) < EXc (−22%) < EXd (−20%) as compared to salinity-stressed control treatment. As in the case of TSS (mg/100 gDW), applied treatments were as EXc (65.3) > EXc + EXd (64.9) = EXd (64.9) in salinity stressed soil, while, under soil conditions, extractants remained as EXd (38.6) > EXc (38.4) > EXc + EXd (37.6). The total protein contents and TFAA showed the same pattern by increasing in stressed plants and decreased significantly with applying algal extracts, i.e., EXc and EXd in alone and combined form (Figure 5A–E).

### 2.6. Enzymatic and Non-Enzymatic Antioxidants

Cellular antioxidants of plants under salinity stress and treated with extractants, such as EXc and EXd, significantly affect the ascorbic acid (AsA, reduced glutathione (GSH), superoxide dismutase activities (SOD), catalase activities (CAT), ascorbic peroxidase (APX) and glutathione reductase (GR). The applied treatments, such as EXc + EXd (88%) > EXd (84%) = EXc (84%), in normal soil, while EXc + EXd (3%) > EXc (2%) > EXc (1%) in saline soil for AsA. A similar trend was observed in the case of CAT. The applied treatments remained as EXc + EXd (370.9) > EXd (369.4) > EXc (364.2) under salinity stress conditions, while, in the normal soil, treatments remained as EXc + EXd (373.6) > EXd (216.1) > EXc (215.8).

**Figure 6:**
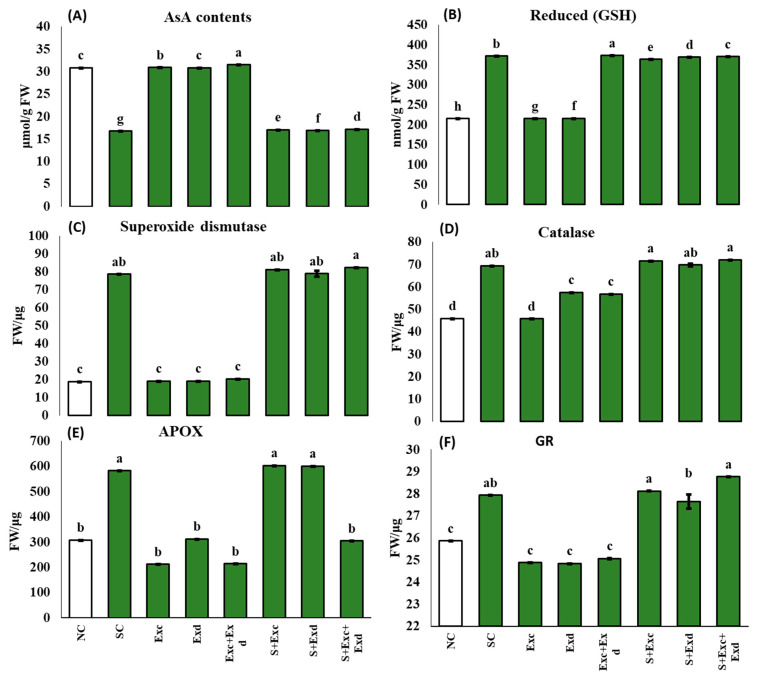
Effect of *Chlorella vulgaris* and *Dunaliella salina* extracts on (**A**) Ascorbic acid (AsA; µmol g^−1^ FW), (**B**) Reduced Glutathione (GSH; nmol/g FW), (**C**) Superoxide dismutase activities (SOD; U/g FW), (**D**) Catalase activities (CAT; U/g FW), (**E**) Ascorbic peroxidase (APX; U/g FW), and (**F**) glutathione reductase (GR; U/g FW) in common bean plants under normal and saline condition soils. On *x*-axis NC; normal soil control, SC; salt-affected soil control, Exc; extract of *Chlorella vulgaris*, Exd; extract of *Dunaliella salina*, Exc + Exd; combined application of *Chlorella vulgaris* and *Dunaliella salina* in normal soil, S + Exc; application of *Chlorella vulgaris* in salt-affected soil, S + Exd; application of *Dunaliella salina* in salt-affected soil, S + Exc + Exd; combined application of *Chlorella vulgaris* and *Dunaliella salina* in salt-affected soil. Bar chart presenting mean values of different treatments, error bars indicating standard deviation, and different lettering on the bars highlighting the significance difference among the applied treatments at a 5% level of significance by applying Duncan’s Multiple Range Test (DMRT).

Other antioxidant enzymes, such as SOD, CAT, APX, and GR, were significantly affected by the application of EXc, EXd, and combined application of EXc and EXd under normal and salinity-stressed conditions. The trend of applied treatments remained as EXc + EXd (5%) > EXc (3%) > EXd (1%) in salt-affected soil, while EXc (−76%) = EXd (−76%) < EX + EXd (−74%) in normal soil for SOD in plants. The CAT was affected by the application EXc + EXd (4%) > EXc (3%) > EXd (1%) under salt-affected soil, while EXc (−34%) < EXc + EXd (−18) < EXd (−17%) in normal soil conditions. The APX and GR were significantly affected by the combined application of EXc and EXd in normal soil conditions and salt-affected soil conditions, as shown in Figure 6.

### 2.7. Mineral Ion Content

**Figure 7:**
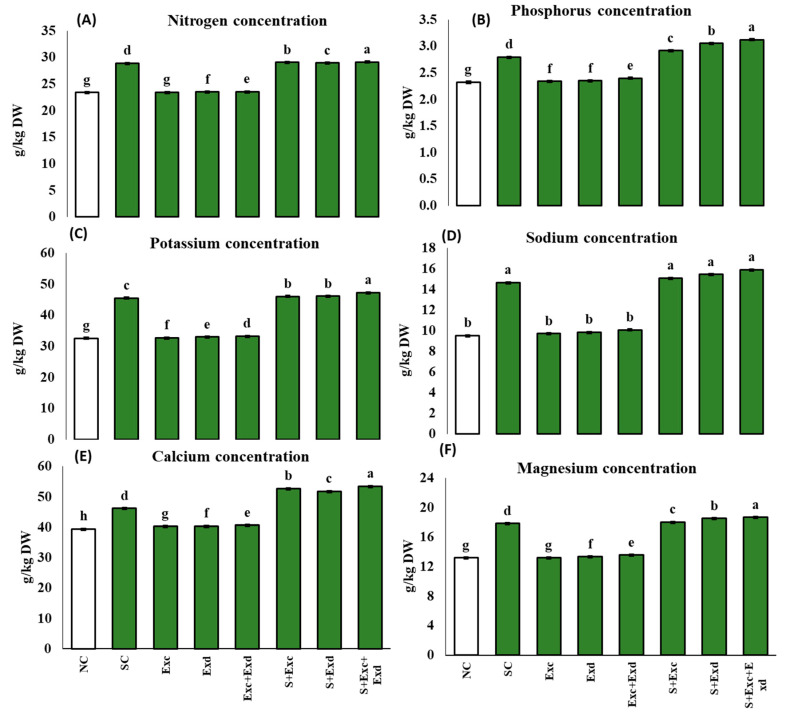
Effect of *Chlorella vulgaris* and *Dunaliella salina* extracts on (**A**) N (g/kg DW), (**B**) P (g/kg DW), (**C**) K (g/kg DW), (**D**) Na (g/kg DW), (**E**) Ca (g/kg DW), (**F**) Mg (g/kg DW) in common bean plants under normal and saline condition soils. On *x*-axis NC; normal soil control, SC; salt-affected soil control, Exc; extract of *Chlorella vulgaris*, Exd; extract of *Dunaliella salina*, Exc + Exd; combined application of *Chlorella vulgaris* and *Dunaliella salina* in normal soil, S + Exc; application of *Chlorella vulgaris* in salt-affected soil, S + Exd; application of *Dunaliella salina* in salt-affected soil, S + Exc + Exd; combined application of *Chlorella vulgaris* and *Dunaliella salina* in salt-affected soil. Bar chart presenting mean values of different treatments, error bars indicating standard deviation, and different lettering on the bars highlighting the significance difference among the applied treatments at a 5% level of significance by applying Duncan’s Multiple Range Test (DMRT).

The concentration of mineral nutrients, such as N, P, K, Ca, Mg, and Na, were significantly affected by the application of extractants, such as EXc and Exd, alone and combined form under normal and salt-affected soil conditions, as shown in Figure 7. In the application of EXc + EXd in salinity stress soil, the concentration of N, P, K, Ca, Mg, and Na was 29.1, 3.1, 47.3, 53.4, 18.7, and 15.9 g/kg of DW, respectively. While under normal soil conditions, the combined application of EXc and EXd, the concentration of N, P, K, Ca, Mg, and Na remained at 23.5, 2.4, 33.2, 40.6, 13.6, and 10.1 g/kg DW, respectively.

### 2.8. Protein Defense Molecules

**Figure 8:**
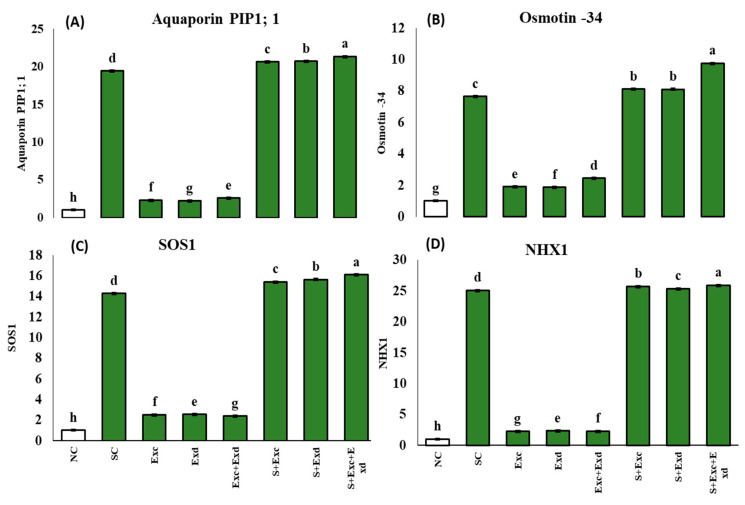
Effect of *Chlorella vulgaris* and *Dunaliella salina* extract on Relative gene expression of (**A**) *PIP1* Aquaporin gene, (**B**) Osmotin-34, (**C**) *SOS1*, (**D**) *NHX1* in common bean plants under normal and saline condition soils. On *x*-axis NC; normal soil control, SC; salt-affected soil control, Exc; extract of *Chlorella vulgaris*, Exd; extract of *Dunaliella salina*, Exc + Exd; combined application of *Chlorella vulgaris* and *Dunaliella salina* in normal soil, S + Exc; application of *Chlorella vulgaris* in salt-affected soil, S + Exd; application of *Dunaliella salina* in salt-affected soil, S + Exc + Exd; combined application of *Chlorella vulgaris* and *Dunaliella salina* in salt-affected soil. Bar chart presenting mean values of different treatments, error bars indicating standard deviation, and different lettering on the bars highlighting the significance difference among the applied treatments at a 5% level of significance by applying Duncan’s Multiple Range Test (DMRT).

The estimated relative gene expression level (*PIP1, osmotin-34, SOS1,* and *NHX1*) in both control, stressed, and treated plants increased in stressed plants (salinity and salinity with extracts) and decreased significantly in plants exposed to algal extracts (Exc, Exd, and both), all showed a highly significant effect induced by salinity (*p* < 0.001), EXc (*p* < 0.001), EXd (*p* < 0.001), the interaction between salinity + EXc (*p* < 0.001), salinity + EXd (*p* < 0.001), as revealed by MANOVA (BM-SPSS version 29.0) (Figure 8). The Poplar aquaporin *PIP1* gene expression in control, salinity-stressed, and treated with algal extracts is presented in Figure 8A. Aquaporin *PIP1* gene showed a constitutive significant overexpression in stressed groups, including salinity and salinity with algal extracts. The *osmotin-34* relative gene expression in control, salinity-stressed, and treated with algal extracts is presented in Figure 8B. Relative expression of *osmotin-34* gene showed a constitutive significant overexpression in stressed groups, including salinity and salinity with algal extracts, where the highest overexpression recorded in group salinity with both extracts (9.754 ± 0.02). The *SOS1* relative gene expression in control, salinity-stressed, and treated with algal extracts is presented in Figure 8C. Relative expression of *SOS1* gene showed a constitutive significant overexpression in stressed groups, including salinity and salinity with algal extracts, where the highest overexpression was recorded in group salinity with both extracts (16.1 ± 0.01). The *NHX1* relative gene expression in control, salinity-stressed, and treated with algal extracts is presented in Figure 8D. Relative expression of *NHX1* gene showed highly significant overexpression in stressed groups, including salinity and salinity with algal extracts, where the highest overexpression was recorded in group salinity with both extracts (25.9 ± 0.01).

**Figure 9:**
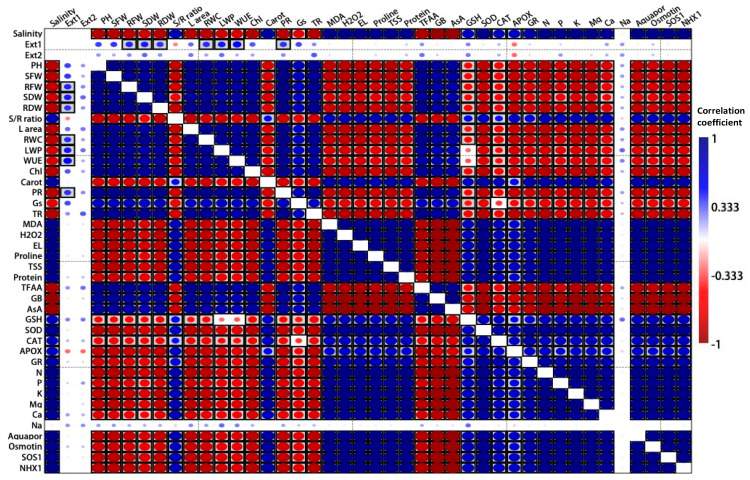
Correlation matrix showing the interaction between different study variables in terms of Pearson’s correlation, where blue color indicates positive correlation, red color for inverse (−) correlation, and boxed colors indicate significant correlation.

**Figure 10:**
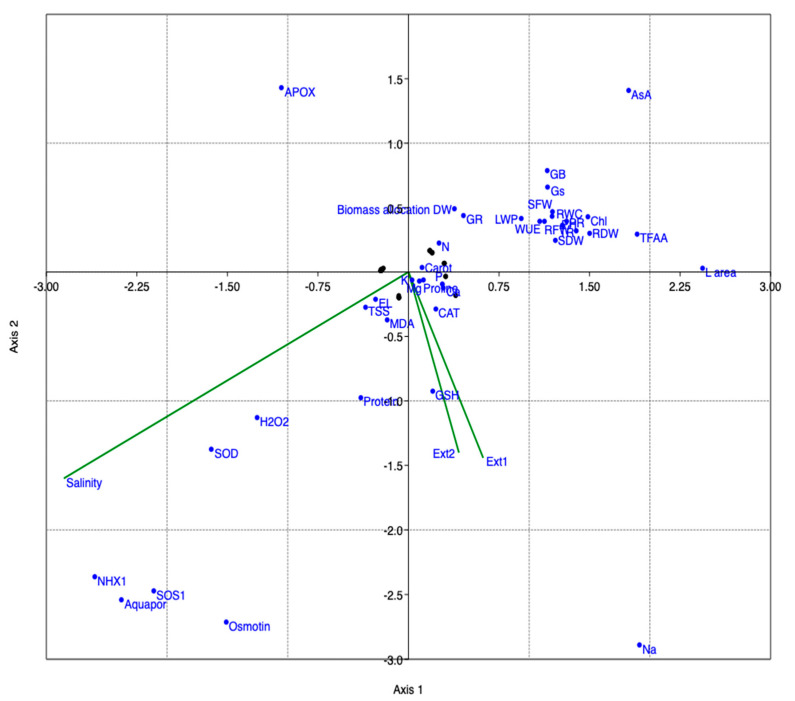
Canonical correspondence analysis showing the effect of salinity, (Ext1 represents EXc; and Ext2 represents EXd) on various variables.

Accordingly, salinity stress showed a highly significant, positive correlation with oxidative damage (MDA, H_2_O_2_, and electrolyte leakage), in addition to proline, total protein, and TSS, however, it inversely significantly correlated with various growth parameters, including (plant height, SFW, RFW, SDW, RDW) in addition to water contents and transpiration rates. EXc (in Figure 9, Ext1 represents EXc; and Ext2 represents EXd) showed a significantly direct positive correlation with growth parameters. Figure 10 represents the canonical correspondence analysis (CCA), which is a multivariate statistical technique used in this experiment to explore the relationships between studied variables. CCA is an extension of correspondence analysis (CA) and canonical correlation analysis (CCA) and is particularly useful in understanding how salinity and applied extractants affect the growth, physiological, biochemical, and nutrient uptake by common bean plants, and the distribution and abundance of species in stressful environments. The CCA ordination represents more than 99% of the total variance induced during the study.

## 3. Discussion

The present study investigated the impact of applying extracts derived from *C. vulgaris* and *D. salina* to common bean plants under salinity stress. In another experiment, an extract of *A. nodosum* was applied to plants in greenhouse experiments conducted within tropical growing conditions [50,51]. The application of this algal extract led to notable results, including the elongation of bean roots and an increase in their levels of essential nutrients, such as potassium, nitrogen, phosphorus, calcium, and magnesium. Moreover, the use of *Chlorella* sp. resulted in an augmentation of available phosphorus and ammonium nitrogen in the soil, ultimately leading to increased pea production. Additionally, *Chlorella* sp., *Spirulina*, *P. palmata*, and *L. digitata* applications in field settings were found to elevate the concentrations of inorganic nitrogen within the soil [52,53]. These heightened elemental concentrations likely played a pivotal role in enhancing plant growth by actively participating in key metabolic functions, such as enzyme activity and protein synthesis [54,55]. The improved growth observed following algal treatment can also be attributed to enhanced antioxidant activity and the accumulation of osmolytes. This is particularly significant as salinity-induced growth decline is primarily driven by reduced cell division and mineral ion leakage, exacerbated by osmotic stress and water deficiency [56]. Encouragingly, the application of AE proved to counteract these negative effects of salinity-induced decline, underscoring the need for further investigations to fully comprehend the underlying mechanisms. The positive growth response of plants treated with seaweed extracts is linked to heightened efficiency in mineral utilization, photosynthesis, and water utilization [57]. Similarly, in a separate study, the treatment of AE on tomatoes resulted in increased growth attributed to enhanced pigment synthesis, improved mineral utilization efficiency, and augmented lipid synthesis [58]. Additionally, the presence of phytohormones such as gibberellins (gibberellic acid), indoles (IAA), and cytokinins (including Trans-Zeatin and Trans-Zeatin riboside) within these extracts is recognized for their regulatory role in plant growth, particularly under stressful conditions [59].

Plants undergoing stress exhibit diminished levels of overall chlorophyll, a phenomenon that can stem from insufficient nutrient absorption [60] and heightened degradation of chlorophyll. A connected study has indicated that specific instances of constrained chlorophyll production, coupled with an upsurge in the chlorophyll-degrading enzyme (chlorophyllase), can result in a decrease in chlorophyll content [61]. Research has documented instances of stress-induced reduction in chlorophyll synthesis [62,63] attributed to a significant decline in the activity of enzymes responsible for chlorophyll production. This reduction is manifested in the levels of chlorophyll intermediates and impacts both the stomatal and non-stomatal aspects of photosynthesis. Furthermore, the occurrence of reactive oxygen species (ROS) during environmental stress conditions leads to the loss of pigments and degradation of chlorophyll, a critical indicator of oxidative harm [64,65]. Consequently, the chlorophyll content in plants serves as a pivotal physiological metric, reflecting the efficacy of photosynthesis [66]. Notably, an elevation in chlorophyll levels as a response to stress can function as a biochemical gauge for a plant’s capacity to endure abiotic stressors [67]. Our study demonstrated that the application of an algal extract substantially elevated chlorophyll levels under optimal conditions and mitigated the detrimental impact of salinity stress. This suggests that the active components within algal extracts confer protection to the chloroplast machinery and enhance the function of chlorophyll-biosynthesizing enzymes. Plants exhibiting heightened chlorophyll synthesis and enhanced mineral absorption experience improved photosynthesis [55,68]. The augmentation of magnesium content also contributes to chlorophyll synthesis, and the decline in chlorophyll and photosynthesis induced by salinity stress can be attributed to significant reductions in magnesium, Rubisco, and chloroplast damage [69].

The heightened pigment content observed in plants treated with algal extracts was associated with a noteworthy improvement in photosynthesis, transpiration, and stomatal conductance. This regulation of stomatal characteristics in *Phaseolus vulgaris* was additionally linked to increased water use efficiency and leaf water potential, consequently influencing plant performance under both normal and stress-induced conditions. Research has indicated that the treatment of seaweed extracts sourced from *Sargassum horneri* can restore growth, chlorophyll content, and photosynthesis in tomato plants [70]. Similarly, the application of seaweed extracts has been reported to enhance photosynthesis, transpiration, and water use efficiency, thereby promoting the growth and sucrose content of sugarcane [57]. Enhanced E, augmented chlorophyll biosynthesis, and improved water potential collectively play a significant role in governing growth and regulating photosynthesis under conditions of salinity stress [17].

Osmoprotectants play a metabolic role in osmotic adjustments, maintaining Relative Water Content (RWC) and MSI in tissues during stress [71,72,73]. Osmolytes play a crucial role in enhancing abiotic stress tolerance in plants [6]. They help to stabilize the osmotic differences between the surroundings of cells and cytosol, which is important for maintaining cellular water balance under stress. Osmolytes also act as compatible solutes, which protect plant cells during osmotic stress situations. Additionally, the extracts provide macro- and micronutrients, bolstering plant defenses against stress impacts [74,75]. Exopolysaccharides found in the extracts contribute to roles, such as electron transport, hormone biosynthesis, membrane fluidity, and protein modification. Osmolytes prevent stress-induced damage by maintaining tissue water, scavenging ROS, and safeguarding enzyme functionality [55].

Proline accumulation during stress triggers osmotic adjustment in plant cells, reducing ROS damage and enhancing stress tolerance [76,77]. Increased proline build-up, possibly due to altered metabolic enzyme activity [54], could have been up-regulated by algal extract treatment. Elevated osmolyte accumulation helps in ROS scavenging and protects vital cellular pathways, such as photosynthesis, thus alleviating stress effects [78,79].

Antioxidants play a pivotal role in counteracting the harmful effects of ROS through both enzymatic and non-enzymatic mechanisms. These mechanisms collectively contribute to the mitigation of ROS, which otherwise could cause damage to crucial molecules such as DNA, proteins, and lipids. This protection is essential in maintaining the optimal growth and functionality of plants [12,49,80]. The effective neutralization of excessive ROS relies on the proper functioning of the antioxidant system. Algal extracts can reduce the production of ROS with their free radical scavenging effect. Algal extracts have antioxidant compounds, such as phenolics, that can scavenge free radicals and protect plants from oxidative damage. The prompt elimination of ROS has a direct positive impact on the functioning of organelles, particularly the chloroplast, ensuring the safeguarding of the photosynthesis process [54]. The heightened antioxidant activity observed in plants treated with algal extracts likely contributes to the preservation of redox homeostasis and the maintenance of the NADP/NADPH ratio. This balance is crucial for protecting electron transport and enzyme functionality. Algal extracts can increase the activity of antioxidant enzymes such as SOD, CAT, and POD in plants [4]. These enzymes play a crucial role in protecting plants from oxidative damage.

Phenolic and flavonoid compounds present in algal sources act as effective scavengers of reactive oxygen species, effectively defending against stress and imparting resistance to salinity-induced stress. Furthermore, flavonoids have the ability to hinder polar auxin transport, leading to localized auxin accumulation in plants [81]. Both enzymatic and non-enzymatic antioxidant activities, including substances, such as proline, soluble sugars, tocopherols, glutathione, and ascorbic acid, play a substantial role in preserving the structural and functional integrity of cellular membranes. This reinforcement of antioxidant mechanisms enhances growth efficiency. Non-enzymatic antioxidants, in conjunction with antioxidant enzymes, demonstrate a significant protective mechanism against environmental stressors, effectively eliminating generated ROS [82,83,84]. Algal extracts possess notable antioxidant properties owing to their rich content of secondary metabolites such as alkaloids, phenolics, terpenoids, and phycobiliprotein pigments, such as phycoerythrin, phycocyanin, and allophycocyanin [85]. By fortifying the antioxidant system’s performance through external supplementation, the detrimental impacts of salinity-induced oxidative damage can be averted. Algal extracts are believed to act as bio-stimulants, providing protective support to bean growth under salinity stress. The heightened antioxidant capabilities of algal extracts are attributed to the abundance of metabolites that effectively scavenge ROS, as previously reported [86]. In wheat, the treatment of extracts derived from *Chlorella* and *Spirulina* has been documented to facilitate the recovery of growth and photosynthesis under salinity-induced stress. This recovery is achieved through the up-regulation of antioxidant enzyme activity [87].

Moreover, essential genes that regulate osmolarity and ion transport in plants were affected by both salinity and algal extract treatment. While salinity led to a noticeable increase, algal extracts further amplified gene expression. Key players, such as *SOS*, *OSMOTIN*, *NHX*, and *PIP,* are pivotal in enhancing salinity tolerance [88,89,90]. Antioxidants, including ascorbate, B-group vitamins, vitamin E, and glutathione, perform crucial roles in biochemical processes that fortify stressed plants against environmental challenges [91]. Recent research highlights the up-regulation of crucial genes, such as *SOS*, *NHX*, *PIP*, and HKT in wheat, reinforcing resistance to salinity stress [17]. The heightened expression of genes encoding transporters significantly aids in containing toxic ions and activating stress signals [92,93]. In this study, the increased expression of the studied genes upon algal extract application likely contributed to improved salinity tolerance by means of stress signaling, salinity exclusion, and maintenance of tissue osmolarity. The collaborative efforts of multiple genes can synergistically elevate stress tolerance, particularly by regulating the balance between Na and K [94]. Experiments with transgenic Arabidopsis lines that overexpress *SOS* and *NHX* have demonstrated enhanced salinity tolerance, reflected in reduced Na levels, increased K levels, and elevated chlorophyll content [95].

Our investigation demonstrated that Ext treatment induced alterations in proline content within stressed plants, further bolstering its accumulation. The presence of amino acids in the extracts triggers the plant’s antioxidant defense system, countering the detrimental effects of stress [91]. Similarly, akin to proline, heightened soluble sugar levels during environmental stress contribute to osmotic adjustments and cellular protection [96,97]. Interestingly, soaking common bean seeds in PrmE or MgE led to heightened proline and soluble sugar concentrations, possibly attributed to the elevated amino acid content in these extracts [91]. Antioxidant parameters, particularly α-tocopherol, uphold cell membrane integrity and alleviate MDA levels by scavenging O^2−^ and OH^−^ radicals, thanks to their electron donation-based antioxidant capability [98,99].

## 4. Materials and Methods

### 4.1. Algal Sources and Extract Preparation

*Chlorella vulgaris* strain (HSSASE3) was identified by accession number (KT277786), and *Dunaliella salina* strain (HSSASE10) was identified by accession number (KT277793), which were acquired from Cairo University, Department of Botany and Microbiology. Axenic *C. vulgaris* was cultivated in modified BG11 medium [100,101], and axenic *D. salina* in F/2 synthetic medium [102]. Both were cultured for 21 days using an orbital shaker (150 rpm), under 150 μmol/m^2^ s PPFD (photosynthetic photon flux density), 24 °C ± 2 temperatures, pH 7, and a 12-h photoperiod. At the stationary growth phase, samples were collected by centrifugation (42,000× *g*, 15 min). Pellets were spread on glass plates, air-dried, and then dried at 50 °C until constant weight was achieved. A foliar spray solution was prepared by dissolving 10 g dried biomass (dried at 50 °C) in 100 mL double distilled water (DW).

### 4.2. Seeds Collection and Environmental Conditions for Plant Growth

*Phaseolus vulgaris* L. Nebraska variety seeds were obtained from the Field Crops Research Institute, Giza, Egypt. Seed surface was sterilized with 2% NaOCl for 5 min, then washed with DDW thrice. Seeds were soaked in 1/4 strength Hoagland solution for 2 h. Ten sterilized seeds were placed in Petri plates with filter paper and 20 mL Hoagland solution. They were germinated in an incubator (PH070A) at 19 °C for 5 days. Healthy seedlings were transplanted to 20 × 15 cm plastic pots with compost and 1.2 kg sterilized sandy loam soil. Growth conditions: natural day/night cycle, day/night temperature of 23/17 ± 3 °C, relative humidity of 65 ± 2%, and photosynthetically active radiation (PAR) of 680 μmol/m^2^s.

### 4.3. Salinity Development and Algal Extract Application

After two weeks (14 DAS) of seed sowing, the number of plants per pot was thinned to one, normal irrigation, and salinity stress was initiated by adding 3000 mg/L NaCl for two weeks (28 DAS). The 15 mL per pot algal extract was applied directly to the soil three times (one every 4 days’ intervals, i.e., at 32, 36, and 40 DAS). The overall experimental treatments were as normal control, salinity stress control (3000 mg/L, NaCl), algal extract of *C. vulgaris* (EXc), algal extract of *D. salina* (EXd), EXc + EXd, NaCl + EXc, NaCl + EXd and NaCl + EXc + EXd. All the treatments were repeated three times, and the experimental design was a complete randomized design (CRD).

### 4.4. Determination of Plant Growth Characteristics and Water-Related Parameters

Plant height was measured using a conventional scale. Green leaf area was determined following the Quarrie protocol [103], calculated with leaf length × leaf width × 0.75 formula. Dry weights were obtained by 70 °C, 24-h oven-drying of root and shoot samples. Relative water content (RWC) was assessed according to the reference [104]. Leaf water potential was assessed between 9:00 and 11:00 AM using a psychrometer on mature leaves with max area and optimal light. Ten measurements were carried out per treatment. Water use efficiency (WUE) was calculated as Pn (net photosynthesis) to Tr (transpiration) ratio after 30 min in darkness. A fluorometer was used on fully developed leaves for 30 min without light [105].

### 4.5. Measurement of Photosynthetic Pigments and Gas Exchange Parameters

Leaf samples (0.2 g) were treated with 10 mL 80% aqueous acetone, then centrifuged (10 min, 12,000× *g*). Using a UV/VIS spectrophotometer (Jenway, Japan), absorbance of the clear solvent was measured at 663 and 645 nm wavelengths. Carotene and chlorophyll contents were determined following Arnon’s method (1949). Photosynthetic gas exchange parameters (net photosynthetic rate, Pn, stomatal conductance, gs, transpiration rate, E) were assessed using a portable infrared gas analyzer (TPS-2, USA) [106]. Measurements were taken on the fifth fully developed leaf from the plant’s top between 9:00 AM and 11:00 AM.

### 4.6. Determination of Oxidative Stress Parameters

The H_2_O_2_ levels were determined by following reference [107]: Fresh leaves were extracted with TCA and centrifuged (15 min, 12,000× *g*). Supernatant (0.5 mL) was mixed with 1 mM potassium iodide and 0.5 mL phosphate buffer (pH 7.0). Absorbance at 390 nm was measured, H_2_O_2_ was calculated using standard curve. The MDA content was measured according to the reference [108]: Fresh leaves were homogenized with TCA and centrifuged (10 min, 10,000× *g*). Supernatant (1 mL) was mixed with 2 mL solution of 0.5% TBA in 20% TCA and boiled 30 min. MDA content was calculated from absorbance difference at 600 and 532 nm after 5 min centrifugation (10,000× *g*). Electrolyte leakage (EL) was assessed by boiling leaf discs in 10 mL of deionized water for EC1 measurement. Tubes were heated at 55 °C for EC2 determination, followed by EC3 measurement after boiling at 100 °C for 10 min, according to reference [109].

### 4.7. Estimation of Osmolytes

Total soluble protein content was determined using Folin Phenol Reagent and Bovine serum albumin as reference, via Bradford’s method at 700 nm absorbance [110]. Total soluble sugars were quantified with anthrone reagent method, absorbance at 625 nm was measured using glucose reference. Free amino acids were estimated using the standard method [111]. GB content in *P. vulgaris* plants, *C. vulgaris*, and *D. salina* algae was calculated via method [112]. Briefly, 0.5 g *P. vulgaris* leaf or microalgae extract was mixed with 10 mL deionized water, homogenized, and combined with 2N H_2_SO_4_. After 2 h ice bath incubation, chilled KI-I2 reagent was added, and tubes were kept at 4 °C overnight. Centrifugation was carried out and betaine periodic complexes were resuspended in 1–2 dichloroethane. Absorbance was measured at 365 nm after 2 h dark settling. Total glycine betaine was calculated using glycine standard curve. Glycine betaine levels were expressed as µg g^−1^ FW. Proline content was measured in bean plants, *C. vulgaris*, and *D. salina* using method [113]. Cells were resuspended in 10 mL (3%, *v*/*v*) sulfosalicylic acid and sonicated. Supernatants were treated with acidic ninhydrin at 80 °C (1 h). Absorbance at 520 nm was measured after complex dissolution in toluene. Proline standard in 3% (*v*/*v*) sulfosalicylic acid was used. Proline levels were reported as µg/g FW.

### 4.8. Assessment of Enzymatic Activity

Fresh *P. vulgaris* leaf (1.0 g) was homogenized in 50 mM pH 7.0 phosphate buffer with 1% polyvinyl pyrrolidine and 1 mM EDTA. Centrifugation (15,000× *g*, 20 min, 4 °C) yielded frozen supernatant [114]. SOD (EC 1.15.1.1) activity was assessed via NBT photochemical reduction at 560 nm after 15 min light incubation [115]. Assay mixture (1.5 mL) contained L-methionine, 75 µM NBT, riboflavin, 50 mM pH 7.5 sodium phosphate buffer, 100 µL EDTA, and 100 µL enzyme extract. SOD activity was expressed as U/mg protein. CAT (EC 1.11.1.6) activity was determined by 240 nm absorbance changes over 2 min [116], using 39.4/mM cm extinction coefficient. APX (EC 1.11.1.11) activity was observed via 290 nm absorbance changes over 3 min with pH 7.0 potassium phosphate buffer, 0.5 mM ascorbic acid, H_2_O_2_, and enzyme extract. Computation used 2.8/mM cm extinction coefficient [117]. GR (EC 1.6.4.2) assay was carried out according to the reference [118]. The assay mixture (1.0 mL) had 100 mL enzyme extract, 0.12 mM NADPH, 0.5 mM GSSG, and 50 mM pH 7.8 sodium phosphate buffer. Absorbance changes at 340 nm were monitored for 2 min, activity was calculated using 6.2/mM cm extinction coefficient, expressed as 1 mol NADPH oxidized/min.

### 4.9. Assessment of Non-Enzymatic Antioxidants

Following [119], ascorbic acid (AsA) was measured by liquid N2-grinding 0.2 g leaf samples, suspending in 2 mL 5% TCA, centrifuging at 10,000× *g* for 15 min at 5 °C. Extraction solution got 10% TCA and was shaken and incubated in ice bath for 5 min. After dilution to 2.0 mL with DDW and adding 0.2 mL diluted Folin–Ciocalteu reagent, the absorbance of the resulting blue color measured at 760 nm after 10 min. For GSH and GSSG measurement in leaf samples, standard protocol [120] was used. Next, 0.4 mL aliquot was neutralized with 0.6 mL 500 mM K phosphate buffer pH 7.0. GSH was determined via NTB absorption at 412 nm due to DTNB reduction. GSSG was measured using 2-vinylpyridine for derivatization and GSH removal.

### 4.10. Mineral Analysis in Plant and Microalgae

For mineral analysis, centrifuged (43,000× *g* for 10 min) and freeze-dried samples of *P. vulgaris* plants, *C. vulgaris*, and *D. salina cultures* (100 mL) were utilized. The Kjeldahl method in microalgae was employed to assess nitrogen content. Total phosphorus estimation was conducted using the ammonium nitro-vanadomolybdate method [121]. Magnesium (Mg), calcium (Ca), and sodium (Na) ion levels were determined through atomic force spectrophotometry (EAA) [122]. Potassium (K) ion concentrations were measured using a flame photometer (Fisher Scientific, Waltham, MA, USA).

### 4.11. Gene Expression

Total mRNA was extracted from 0.25 g *P. vulgaris* leaves using Sigma-Aldrich RNA kit. Purified RNA was quantified and evaluated on 1% agarose gel. Reverse transcription was carried out with oligo dT primer, buffer, MgCl_2_, dNTPs, reverse transcriptase, RNA. RT-PCR at 42 °C for 1 h, 72 °C for 20 min. Real-time PCR utilized SYBR^®^ Green, gene-specific primers, reference genes (β-Actin, GAPDH) on Rotor-Gene 6000. Reactions in 20 µL were carried out with template, SYBR Green Master Mix, primers, DW. PCR: 95 °C for 15 min, then 40 cycles of 95 °C for 30 s, 60 °C for 30 s. ΔCT was calculated calculated (target gene CT minus β-Actin gene CT), gene expression was determined using 2^−ΔΔCt^ method [123]. GAPDH (Glyceraldehyde-3-phosphate dehydrogenase) was used as housekeeping gene expression for gene analysis (Table 2).

### 4.12. Data Analysis

Data normality was assessed using the Shapiro–Wilk test to determine parametric or nonparametric characteristics. The reported values represent the mean of three replicates, accompanied by standard error calculations. Significance levels were determined through one-way ANOVA followed by DMRT. IBM-SPSS version 29.0 for Mac OS was utilized for all data analyses.

## 5. Conclusions

In conclusion, the study highlights the significant benefits of applying algal extracts from Chlorella vulgaris and *Dunaliella salina* to *Phaseolus vulgaris* (common bean) plants. These benefits encompass enhanced growth and photosynthesis, increased mineral content crucial for plant development, improved antioxidant systems guarding against oxidative stress, and a potential influence on genetic mechanisms regulating growth and stress responses. Moreover, the study underscores the promising role of algal extracts in mitigating the detrimental effects of salinity on plant growth and oxidative damage. While these findings offer valuable insights into the potential of algal extracts as plant supplements, they also emphasize the need for further research to comprehensively unravel the intricate biochemical and genetic processes responsible for these observed effects. Such additional research is essential to fully harness the capabilities of algal extracts for improving the performance and resilience of common bean plants and potentially other crops facing similar challenges.

## Figures and Tables

**Table 1:** Biochemical characteristics of the applied microalgae, such as *Chlorella vulgaris* and *Dunaliella salina*.

Biochemical Parameter	*Chlorella vulgaris*	*Dunaliella salina*	Independent *t*-Test	
	Mean ± SD	Mean ± SD	T	*p*-Value
Total soluble protein (mg/g DW)	48 ± 0.43	37 ± 0.23	39.1	<0.001 ***
Total soluble sugar (mg/g DW)	68 ± 0.39	73 ± 0.33	−17.0	<0.001 ***
Proline (µg/g FW)	39 ± 0.24	52 ± 0.33	−55.2	<0.001 ***
Glycine betaine (µg/g FW)	65 ± 0.18	78 ± 0.36	−55.9	<0.001 ***
Nitrogen (mg/g DW)	15 ± 0.12	10 ± 0.35	23.4	<0.001 ***
Phosphorus (mg/g DW)	9 ± 0.81	6 ± 0.26	6.1	<0.002 **
Potassium (mg/g DW)	12 ± 0.39	9 ± 0.51	8.1	<0.001 ***
Magnesium (mg/g DW)	6 ± 0.28	3 ± 0.22	14.6	<0.001 ***
Sodium (mg/g DW)	15 ± 0.03	12 ± 0.40	13.0	<0.001 ***

**, significant at <0.05 but greater than 0.001 of *p*-value, ***, highly significant difference at <0.001 of *p*-value.

**Table 2:** Primers used for gene expression studies.

Primer Name		Sequence
*NHX1*	F	5′-CTCAAGGGTGACTACCAAGCA-3′
	R	5′-CCAATGCATCCATCCCGAC-3′
*SOS1*	F	5′-GAATCAAATCCTAGTNACGCCG-3′
	R	5′-GAATCAAATCCTAGTNACGCCG-3′
OSMOTIN (OSM34)	F	5′-CTCTCAACACGTTTGGACATTGTC-3′
	R	5′-TTGAACCAATTCAACAACTTAGAC-3′
aquaporins *PIP1*; 1	F	5′-GATTGGGAGCTAACAAATTCAACG-3′
	R	5′-CTGCAATACCAGCCCTGTAAAAAG-3′
GAPDH	F	5′-TTGGTTTCCACTGACTTCGTT-3′
	R	5′-CTGTAGCCCCACTCGTTGT-3′
β-Actin	F	5′-TGCATACGTTGGTGATGAGG-3′
	R	5′-AGCCTTGGGGTTAAGAGGAG-3′

## Data Availability

The data presented in this study are available on request from the corresponding author.
